# Effects of Intense Pulsed Light (IPL) Rapid Annealing and Back-Channel Passivation on Solution-Processed In-Ga-Zn-O Thin Film Transistors Array

**DOI:** 10.3390/mi11050508

**Published:** 2020-05-18

**Authors:** Hyun Jae Kim, Chul Jong Han, Byungwook Yoo, Jeongno Lee, Kimoon Lee, Kyu Hyoung Lee, Min Suk Oh

**Affiliations:** 1 Display Research Center, Korea Electronics Technology Institute (KETI), Seongnam, Gyeonggi-do 13509, Korea; sotree@keti.re.kr (H.J.K.); cjhan@keti.re.kr (C.J.H.); bwyoo@keti.re.kr (B.Y.); jnlee123@keti.re.kr (J.L.); 2 Department of Materials Science and Engineering, Yonsei University, Seoul 03722, Korea; khlee2018@yonsei.ac.kr; 3 Department of Physics, Kunsan National University, Gunsan, Jeollabuk-do 54150, Korea; kimoon.lee@kunsan.ac.kr

**Keywords:** thin film transistor, In-Ga-Zn-O (IGZO), solution process, intense pulsed light (IPL), passivation

## Abstract

We report on the effects of the intense pulsed light (IPL) rapid annealing process and back-channel passivation on the solution-processed In-Ga-Zn-O (IGZO) thin film transistors (TFTs) array. To improve the electrical properties, stability and uniformity of IGZO TFTs, the oxide channel layers were treated by IPL at atmospheric ambient and passivated by photo-sensitive polyimide (PSPI). When we treated the IGZO channel layer by the IPL rapid annealing process, saturation field effect mobility and subthreshold swing (S.S.) were improved. And, to protect the back-channel of oxide channel layers from oxygen and water molecules, we passivated TFT devices with photo-sensitive polyimide. The IGZO TFTs on glass substrate treated by IPL rapid annealing without PSPI passivation showed the field effect mobility (*μ*_FE_) of 1.54 cm^2^/Vs and subthreshold swing (S.S.) of 0.708 V/decade. The PSPI-passivated IGZO TFTs showed higher *μ*_FE_ of 2.17 cm^2^/Vs than that of device without passivation process and improved S.S. of 0.225 V/decade. By using a simple and fast intense pulsed light treatment with an appropriate back-channel passivation layer, we could improve the electrical characteristics and hysteresis of IGZO-TFTs. We also showed the improved uniformity of electrical characteristics for IGZO TFT devices in the area of 10 × 40 mm^2^. Since this IPL rapid annealing process could be performed at a low temperature, it can be applied to flexible electronics on plastic substrates in the near future.

## 1. Introduction

To satisfy the requirements for the next generation display such as large area, high resolution and high frame rate, and other applications such as sensing, signal amplification, signal processing and RFID tags, thin film transistor technologies using oxide semiconductors have been investigated by many researchers and engineers very actively, due to large-area uniformity, low off current and high field effect mobility [1,2,3,4,5,6,7,8,9,10,11,12,13,14,15]. For flexible display applications, pixel-switching/driving transistors and light emitting devices should be fabricated on flexible substrates. However, most of the flexible substrates for display applications are polymeric substrates, such as polyethylene terephthalate (PET), polyethylene naphthalate (PEN) and polyimide (PI) [16,17,18]. Therefore, we have to develop low-temperature fabrication process to minimize damages on substrates, and misalign problems caused by thermal expansion and shrinkage during fabrication process.

Until now, the conventional fabrication processes for oxide thin film transistors (TFTs) in the real industrial applications are thin film deposition by radio frequency (RF) magnetron or direct current (DC) sputtering in vacuum chamber systems and thermal annealing at higher temperature than 300 °C in clean dry air (CDA) ambient for about 1 h [19]. To decrease the process temperature for flexible systems, there were several reports about solution-processed oxide TFT at low temperature [20,21,22,23]. However, those methods have several disadvantages: (1) it needs oxygen and humid free ambient [21]; (2) combustion reaction can make the film porous [22]; and (3) it takes a long time: more than 1 h 30 min [23]. So, to decrease the fabrication time in ambient conditions and make dense channel layers, we developed a rapid post-annealing process for solution-processed oxide channel layer at low temperature by the intense pulsed light (IPL) process. Up to now, there have been several reports about solution-processed oxide TFTs by the IPL process [24,25,26,27,28,29,30,31]. However, there were no results related to the uniformity of oxide thin film transistors for large area display application. To improve the stability of devices, there was a report on top gated solution processed InGaZnO TFTs with light pulse annealing, where the channel was covered with different polymers, which can also act as passivation layers and gate dielectrics simultaneously [31]. Therefore, based on these pervious reports, we report on the feasibility of the IPL rapid annealing process for multi-component oxide thin film transistors, and the improved uniformity and hysteresis properties of electrical performances for 18 IGZO TFT devices in the area of 10 × 40 mm^2^.

## 2. Experimental Procedure 

**Figure 1:**
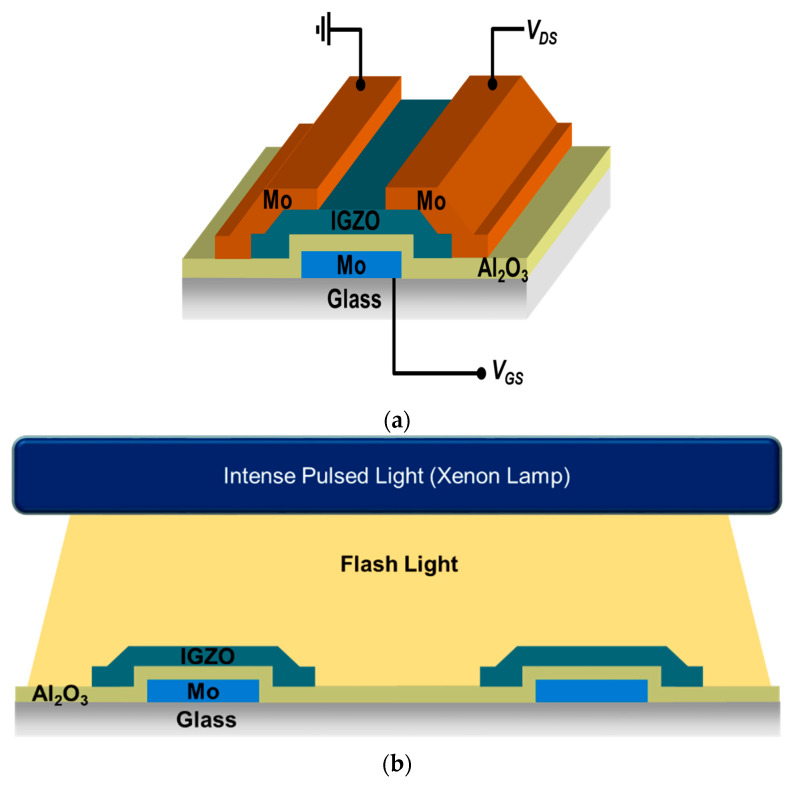
Schematic diagrams of (**a**) In-Ga-Zn-O (IGZO) thin film transistor device with inverted-staggered structure and (**b**) intense pulsed light (IPL) rapid annealing system with xenon flash lamp.

To fabricate IGZO oxide thin film transistors array by intense pulsed light (IPL) process, we fabricated our devices on Si wafer and glass substrate. These substrates were cleaned by ultrasonic cleaner with acetone, methanol and isopropyl alcohol (IPA). In case of Si wafer, we used thermally-grown SiO_2_ (200 nm) as gate insulator on highly-doped p-Si wafer (0.001~0.005 Ω·cm) as a gate electrode. And we also fabricated devices on glass substrates coated with molybdenum (Mo). For the gate electrode of TFTs, we patterned Mo layer by conventional photolithography and wet etching process. After gate patterning, we deposited an Al_2_O_3_ layer of 140 nm as a gate insulator by atomic layer deposition (ALD), using trimethylaluminium (TMA) and H_2_O at 300 °C on gate-patterned Mo glass. The substrates with ALD-grown Al_2_O_3_ were treated by O_2_ plasma (150 W) for 60 s. Then, to deposit the In-Ga-Zn-O (IGZO) channel layer on the gate insulator, we prepared metal precursors and solvent. After indium nitrate hydrate (In(NO_3_)_3_·*x*H_2_O, 0.1104 M), gallium nitrate hydrate (Ga(NO_3_)_3_·*x*H_2_O, 0.0125M) and zinc acetate di hydrate (Zn(CH_3_CO_2_)_2_·2H_2_O, 0.0275 M) were dissolved in 2-methoxy ethanol (2ME), the solution was stirred at 50 °C and spin-coated for 6 s at 500 rpm and 60 s at 3000 rpm on substrate. The IGZO-coated substrate was pre-annealed on a hot plate (H/P) at 300 °C for only 10 min, to minimize the effects of residual solvent and maximize the differences, according to the IPL process conditions for multi-component IGZO channel layers. To activate channel layer electrically, IGZO layer were irradiated by the xenon flash lamp (XENON Corporation, Wilmington, DE, USA, Z-1000) of IPL system with the pulse width of 1~2 ms. The distance between xenon lamp and substrate was 3 cm. Then source/drain electrodes were deposited by dc magnetron sputtering (150 W, Ar only) at room temperature, and patterned by the lift-off process with the positive photoresist and poly(methyl methacrylate) (PMMA, MicroChem C4 or A4) double layer. For the passivation of TFT array, PSPI (Photo-Sensitive Polyimide, PICOMAX, PSPI 1000P, Seoul, Korea) was spin-coated on the IGZO TFT devices. After exposure and a developing processes, the hard baking process of PSPI-coated devices was carried out at 230 °C. Figure 1a shows the schematic diagram of our basic device structure with a bottom gate. 

After the optimization of fabrication process, we fabricated the IGZO TFTs array with the active area of 10 × 40 mm^2^ to evaluate the uniformity of TFTs. Current-voltage (I–V) characteristics were measured by a semiconductor parameter analyzer (HP4156C, Agilent Technologies, Santa Clara, CA, USA) at room temperature in the dark, and an air ambient with the relative humidity of 30%. The saturation field effect mobility was calculated from transfer curves by following equation,

(1) $$I_{D}=\frac{C_{ox}\mu_{n}W}{2L}{\left(V_{G}-V_{th}\right)}^{2}$$

where *C_ox_* is the capacitance of gate insulator, *W* is the width of source/drain electrode, *L* is the length between source and drain electrode, and *V_th_* is the threshold voltage).

To analyze chemical bonding in the IGZO channel layer, the binding energies of oxygen were measured by X-ray photoelectron spectroscopy (XPS, Thermo Scientific, K-alpha, Waltham, MA, USA). And, to observe the cross section of IGZO channel layers, we measured high resolution transmission electron microscopy (HRTEM, JEOL, JEM-2100F, Tokyo, Japan).

## 3. Results and Discussion

To analyze various effects of intense pulsed light (IPL) process on solution-processed IGZO oxide TFT devices, we chose the inverted-staggered structure with a bottom-gate configuration (Figure 1a). Figure 1b shows a schematic diagram of IPL process with xenon flash lamp on In-Ga-Zn-O channel layers. Before source/drain electrodes were deposited, we annealed IGZO oxide channel layers by IPL rapid process at room temperature in air ambient to observe the effects only on oxide channel layers in TFT devices without those related with source/drain (S/D) electrodes. The distance between a xenon flash lamp and IGZO channel layer was 3 cm. To minimize the effects of residual solvent and compare various properties of devices by voltage conditions and shot numbers effectively and clearly, we annealed our devices by IPL process after the pre-annealing on hotplate at 300 °C for 10 min. When our IGZO channel layers on Si wafer were treated by IPL process, we used 2 and 3 kV of input voltage with 10 and 15 shots, to compare the effects of input voltages and shot numbers. To compare effects according to transferred energy from xenon lamp, we used a pulse width of 1 ms for 2 kV (shorter pulse, lower power) and 2 ms for 3 kV (longer pulse, higher power). When we measured transfer curves of each device, the range of gate voltage was from −20 V to 30 V, and the drain voltage was 30 V. 

**Figure 2:**
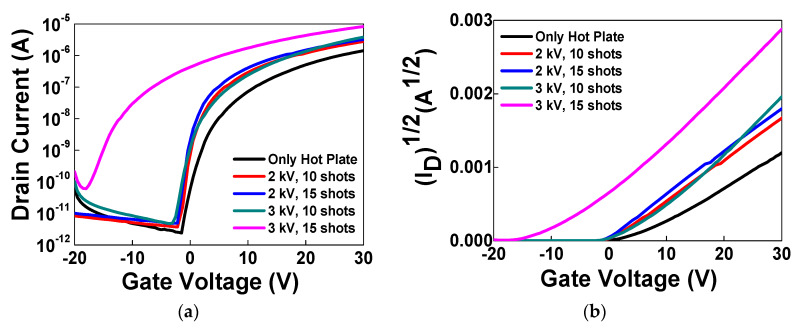
(**a**) The drain current (logarithmic scale) versus gate voltage (*V_G_*) plots and (**b**) the square root of drain current versus gate bias (√*I_D_*–*V_G_*) transfer curves of IGZO thin film transistors (TFTs) on Si wafer annealed on only hot plate and with additional IPL processes with 2~3 kV and 10~15 shots, as obtained under a drain bias (*V_D_*) of 30 V. (Pulse widths: 1 ms for 2 kV, 2 ms for 3 kV).

The field effect mobility and drain current of the device on Si wafer, which was annealed by IPL with 2 kV and 10 shots, was improved in comparison to the device that was annealed only on a hot plate (from 1.79 × 10^−2^ cm^2^/Vs to 2.75 × 10^−2^ cm^2^/Vs). In the case of the 2 kV condition, drain current and subthreshold swing value of device with 15 shots was better than those of the device with 10 shots. However, the device with 2 kV and 1 ms showed a lower field effect mobility (3.01 × 10^−2^ cm^2^/Vs) than those (>4 × 10^−2^ cm^2^/Vs) of the devices which were annealed by IPL with 3 kV and 2 ms. In case of IPL process with 3 kV, the device with 15 shots showed a negative shift of threshold voltage from 5.1 V to −6.5 V (Figure 2b) and the off current increased (Figure 2a). From these results, we could know that the optimized irradiation conditions of IPL process can improve the properties of IGZO oxide TFT devices, but excessive irradiation can cause deterioration of channel layers. 

**Figure 3:**
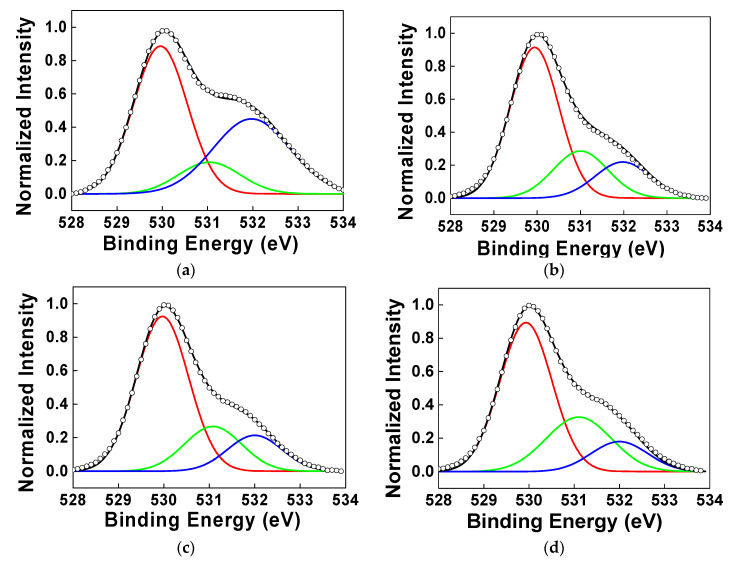
The X-ray photoelectron spectroscopy (XPS) spectra (black lines with open circle symbols) of (**a**) hot plate (H/P) at 50 °C for 2 min, (**b**) H/P at 300 °C for 10 min, (**c**) H/P + IPL (3 kV, 10 shots) and (**d**) H/P + IPL (3 kV, 15 shots). The data shows the peak position of O1s. Deconvolution of the XPS spectra shows the contributions of peaks at 529.9 (red), 531.0 (green) and 531.9 eV (blue) from oxygen atoms, respectively, in M–O–M lattice, near oxygen vacancies and in M–OH compounds.

To analyze the reason for these phenomena, X-ray photoelectron spectroscopy (XPS) measurement was carried out. Figure 3 shows the O1s XPS spectra for (a) hot plate (H/P) at 50 °C for 2 min, (b) H/P at 300 °C for 10 min, (c) H/P + IPL (3 kV, 10 shots) and (d) H/P + IPL (3 kV, 15 shots), respectively. To compare with the reference sample, we fabricated a reference IGZO channel layer which was annealed at 50 °C for drying solvent after solution coating. In the XPS data, when we divided the oxygen peak into three Gaussian components with variable full-widths at half-maximum (FWHMs), we could know that the peaks centered around 529.9, 531.0 and 531.9 eV could reflect three different oxygen environments [21,23,32]. The peaks at 529.9 eV are attributed to oxygen atoms in M–O–M lattice, and the peaks at 531.0 eV are attributed to oxygen atoms near oxygen vacancies. The peaks at 531.9 eV are attributed to oxygen atoms in M–OH compounds. When we annealed IGZO channel layer only at 50 °C, there were many residual organic components from metal precursors. Therefore, relatively more M-OH bondings remained (Figure 3a), and the peak intensity at 531.9 eV became larger than that of the peak at 531.0 eV by more than two times. In the case of annealing at 300 °C, these organic residues from precursor decreased. Therefore, the peak at 531.9 eV decreased, compared to the peak related with M-O-M bondings at 529.9 eV. When we used 10 shots in IPL process, M-OH bonding decreased, due to the decomposition and removal of organic residues from metal precursors caused by irradiation of xenon flash lamp. In case of 15 shots, more oxygen vacancies were generated, because larger energy from xenon lamp could break bondings between oxygen and metal and remove oxygen atoms from the IGZO layers more easily. Generally, ZnO-based oxide semiconductor materials show n-type conductive behavior because of native defects, such as oxygen vacancies, cation interstitials and hydrogen. If there are oxygen vacancies in IGZO oxide channel layers, n-type carriers (electrons) caused by metal cation can be charge carriers, which can function as a shallow donor and enhance the conductivity of oxide semiconductors [33]. For this reason, the IGZO TFT device annealed with 15 shots of IPL process showed conductive properties in the transfer curve (Figure 2).

**Figure 4:**
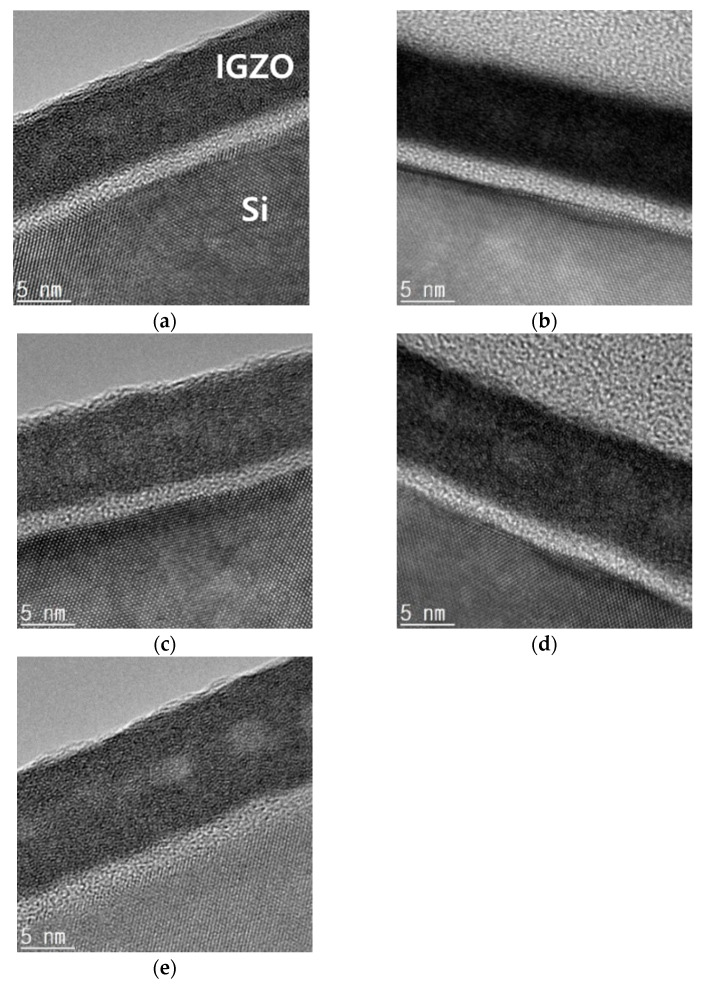
Cross-section high resolution transmission electron microscopy (HRTEM) images of IGZO films on Si wafers annealed by (**a**) deep UV photochemical activation; thermal annealing at 350 °C for (**b**) 20 and (**c**) 100 min; intense pulsed light (IPL) annealing width pulse width of 2 ms and (**d**) 10 and (**e**) 15 shots.

To confirm the degree of film densification of oxide channel layers with various annealing conditions by measuring thickness, we carried out cross-sectional HRTEM analysis (Figure 4). For TEM measurement, we coated IGZO solution directly on p-type Si wafers. To compare our IPL-treated samples with other annealing processes, such as deep UV and thermal annealing processes [23], we additionally fabricated various IGZO oxide layers annealed by deep UV photochemical activation processes for 2 h, and hot plate at 350 °C for 20 and 100 min. Based on cross-section TEM images, the thicknesses of these three layers with various annealing processes were about 8.7~8.8 nm, and the thicknesses of oxide channel layers which were fabricated by hot plate and IPL annealing with pulse width of 2 ms and 10 and 15 shots were about 10~10.5 nm. Although the thicknesses of the IPL-treated oxide layers are slightly thicker than others, there are no big differences among five oxide layers. Therefore, we could know that the densities of IGZO layers with IPL treatments are similar to previous UV- and thermal-treated layers, and the IPL annealing process can be applied to the oxide TFT fabrication process, instead of the conventional annealing process. 

For the applications to active matrix display panel and image sensor array, we fabricated 10 × 40 IGZO TFTs array on gate-patterned Mo glass substrate with an ALD-grown Al_2_O_3_ gate insulator, based on the IPL annealing process conditions. When we fabricated TFT devices on Si wafer, the conditions of 3 kV, 15 shots and a pulse width of 2 ms showed conductive transfer characteristics. However, because the Si wafer and glass substrate have different characteristics under IPL irradiation, we fabricated two kinds of TFT devices on the glass substrate with different IPL conditions. One was a IGZO TFTs array with a shorter pulse width (irradiation time), and the other was a IGZO TFTs array with fewer shots. We had tested various IPL process conditions and selected two process conditions: (3 kV, pulse width of 1 ms: 323 mJ/cm^2^ × 15 shots) and (3 kV, pulse width of 2 ms: 638 mJ/cm^2^ × 10 shots). Generally, oxide TFTs can be degraded by the adsorption of oxygen or water molecules on the back-channel surface of oxide semiconductor [34,35]. Therefore, to reduce the instability of IGZO TFT devices, we additionally applied polyimide as a passivation layer to IGZO TFTs [36]. Polyimide can endure a higher process temperature than other polymer passivation materials and, in the case of photosensitive polyimide (PSPI), it is easy to make a via-hole for source/drain electrodes contact, so we used PSPI as the passivation layer for our IPL-treated IGZO TFT devices.

**Figure 5:**
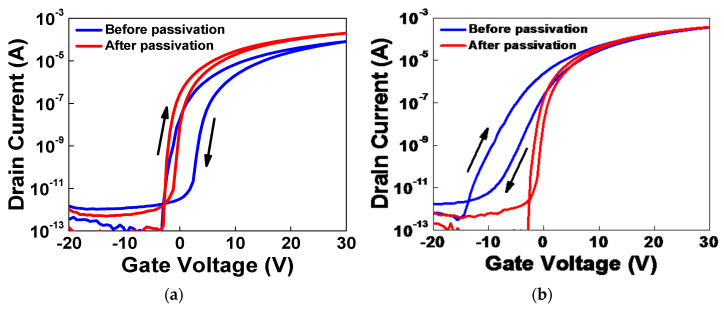
Log_10_(*I_D_*) *vs.* *V_G_* transfer curves of IGZO TFTs on glass substrate treated by the IPL annealing process before and after photosensitive polyimide (PSPI) passivation. (**a**) 3 kV, pulse width of 1 ms, 15 shots; (**b**) 3 kV, pulse width of 2 ms, 10 shots.

In Figure 5, both cases showed improved hysteresis after PSPI passivation. In Figure 5a, IGZO TFT device was treated by IPL annealing process with 3kV, pulsed width of 1 ms and 15 shots. The saturation field effect mobility was improved from 0.44 to 1.13 cm^2^/Vs and subthreshold swing (S.S.) decreased from 0.24 to 0.19 V/decade. In Figure 5b, the IGZO TFT device was treated by IPL annealing process with 3kV, 2 ms, 10 shots. The saturation field effect mobility was improved from 1.58 to 2.23 cm^2^/Vs and subthreshold swing (S.S.) decreased from 1.06 to 0.18 V/decade. Although S.S. values are similar to each other, we selected the latter to fabricate devices and evaluate electrical properties and uniformity of the IGZO TFTs array treated by the IPL annealing process, because the mobility in Figure 5b is higher than that in Figure 5a.

**Figure 6:**
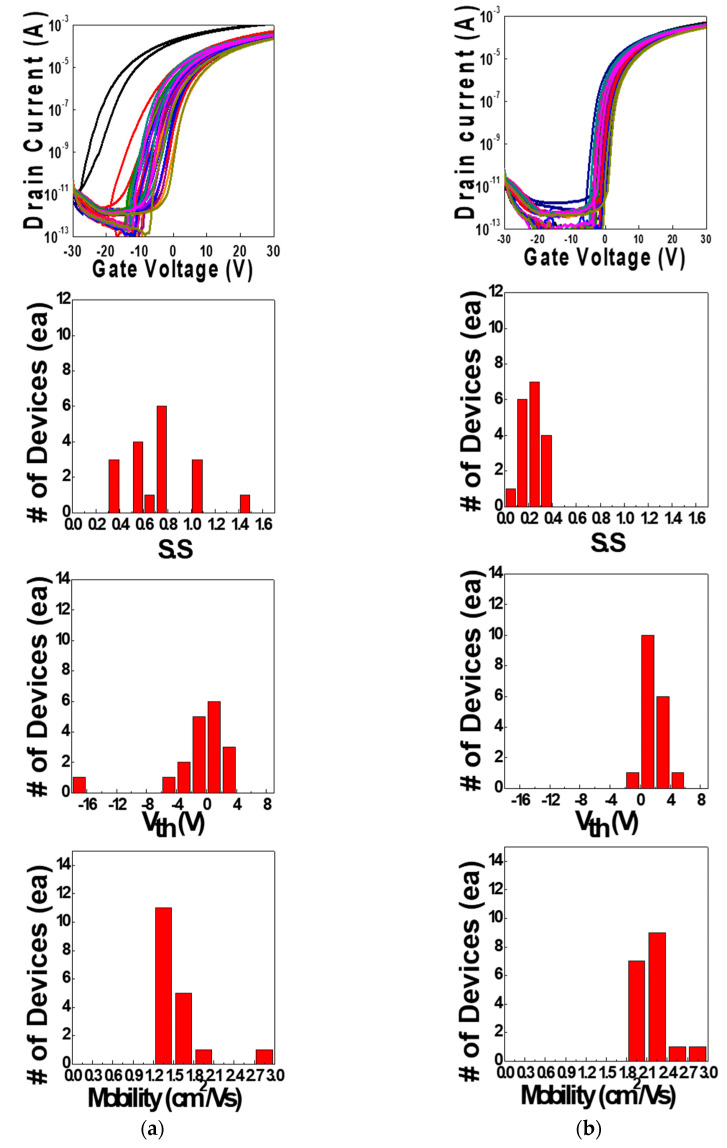
Log_10_(*I_D_*) vs. *V_G_* transfer curves and the statistical data of subthreshold swing (S.S.) values, threshold voltages (*V_th_*) and saturation field effect mobilities (**a**) before and (**b**) after PSPI-passivation.

Figure 6 shows the log_10_(*I_D_*) vs. *V_G_* transfer curves and the statistical data of IGZO TFTs array treated by the IPL annealing process (a) before and (b) after passivation. We measured the electrical characteristics of 18 TFTs before and after PSPI passivation. In Figure 6a, the IGZO TFT devices without passivation showed non-uniform electrical characteristics. However, the TFT devices with passivation showed more uniform properties with Gaussian-like distribution. From these data, we could know that the electrical characteristics and uniformities of the IPL-treated and PSPI-passivated IGZO TFTs were better than those of the unpassivated devices. The important electrical characteristics of these IGZO TFT devices are listed in Table 1. The average saturation field effect mobility (*μ*_FE_) and subthreshold swing (S.S.) for the unpassivated IGZO ZTO TFTs were 1.54 cm^2^/Vs and 0.708 V/decade, respectively. In case of passivated devices, the average u_FE_ and S.S. were 2.17 cm^2^/Vs and 0.225 V/decade, respectively. We could know that the uniformities and standard deviations of the electrical properties with PSPI-passivation were improved, compared to the devices without passivation. From these data, we could conclude that the IPL post-annealing treatment and PSPI passivation process gave positive effects to the IGZO TFT devices.

## 4. Conclusions

We fabricated solution-based IGZO TFT devices with inverted-staggered/bottom-gate structure and improved the electrical properties, stability and uniformity of TFTs array. To improve the various properties of IGZO TFTs array, the oxide channel layers were treated by IPL annealing at atmospheric ambient and the back-channel surfaces of IGZO channel layers were passivated by photo-sensitive polyimide (PSPI). When we treated IGZO channel layer by the IPL rapid annealing process, saturation field effect mobility and subthreshold swing (S.S.) were improved. To protect the back-channel of oxide channel layers from oxygen and water molecules, we passivated TFT devices with photo-sensitive polyimide. The IGZO TFTs treated by IPL rapid annealing without PSPI passivation showed the average field effect mobility (*μ*_FE_) of 1.54 cm^2^/Vs, and a subthreshold swing (S.S.) of 0.708 V/decade. The PSPI-passivated IGZO TFTs showed a higher average *μ*_FE_ of 2.17 cm^2^/Vs than that of the device without passivation process and improved S.S. of 0.225 V/decade. When the back-channel surface of IGZO channel layers were passivated by PSPI, the saturation field effect mobilities (u_FE_), the subthreshold swing (S.S.) and the uniformity of IGZO TFT devices were improved. So, we can conclude that we could drastically improve the electrical properties, stability and uniformity of IGZO TFTs array by using a simple and fast IPL treatment with an appropriate back-channel passivation layer, which can be used for the large area process. Since this IPL rapid annealing process could be performed at low temperature, it can be applied to flexible electronics on plastic substrates in the near future. 

## Figures and Tables

**Table 1:** The average values, uniformities and standard deviations of saturation mobilities (*μ*_FE_) and subthreshold swings (**a**) before and (**b**) after PSPI-passivation are shown. The uniformity values were calculated by [(maximum − minimum)/(2·average)] × 100.

Parameters	Before Passivation			After Passivation		
	Average	Uniformity (%)	St. Dev.	Average	Uniformity (%)	St. Dev.
*μ*_FE_ (cm^2^/Vs)	1.54	48.8	0.33	2.17	18.7	0.19
S.S (V/decade)	0.708	75.5	0.28	0.225	57.8	0.07

## References

[1] Nomura K., Ohta H., Takagi A., Kamiya T., Hirano M., Hosono H. (2004). Room-temperature fabrication of transparent flexible thin-film transistors using amorphous oxide semiconductors. Nature.

[2] Wager J.F. (2016). Oxide TFTs: A Progress Report. J. Inf. Disp..

[3] Park S.-H.K., Hwang C.-S., Ryu M., Yang S., Byun C., Shin J., Lee J.-I., Lee K., Oh M.S., Im S. (2009). Transparent and Photo-stable ZnO Thin-film Transistors to Drive an Active Matrix Organic-Light- Emitting-Diode Display Panel. Adv. Mater..

[4] Oh M.S., Lee K., Lee K.H., Cha S.H., Choi J.M., Lee B.H., Sung M.M., Im S. (2009). Transparent Photo-Stable Complementary Inverter with an Organic/Inorganic Nanohybrid Dielectric Layer. Adv. Func. Mater..

[5] Hayashi R., Ofuji M., Kaji N., Takahashi K., Abe K., Yabuta H., Sano M., Kumomi H., Nomura K., Kamiya T. (2007). Circuits using uniform TFTs based on amorphous In-Ga-Zn-O. J. Soc. Inf. Disp..

[6] Barquinha P., Lereira L., Goncalves G., Martins R., Fortunato E. (2009). Toward High-Performance Amorphous GIZO TFTS. J. Electrochem. Soc..

[7] Nomura K., Takagi A., Kamiya T., Ohta H., Hirano M., Hosono H. (2006). Amorphous Oxide Semiconductors for High-Performance Flexible Thin-Film Transistors. Jpn. J. Appl. Phys..

[8] Lee J., Lee J., Park J., Lee S.-E., Lee E.G., Im C., Lim K.-H., Kim Y.S. (2019). Solution-Grown Homojunction Oxide Thin-Film Transistors. ACS Appl. Mater. Interfaces.

[9] Cai J., Han D., Geng Y., Wang W., Wang L., Zhang S., Wang Y. (2013). High-Performance Transparent AZO TFTs Fabricated on Glass Substrate. IEEE Trans. Electron Devices..

[10] Ong B.S., Li C., Li Y., Wu Y., Loutfy R. (2007). Stable, Solution-Processed, High-Mobility ZnO Thin-Film Transistors. J. Am. Chem. Soc..

[11] Lee S., Nathan A. (2016). Subthreshold Schottky-barrier thin-film transistors with ultralow power and high intrinsic gain. Science.

[12] Knobelspies S., Daus A., Cantarella G., Petti L., Münzenrieder N., Tröster G., Salvatore G.A. (2016). Flexible a-IGZO Phototransistor for Instantaneous and Cumulative UV-Exposure Monitoring for Skin Health. Adv. Electron. Mater..

[13] Pecunia V., Banger K., Sou A., Sirringhaus H. (2015). Solution-based self-aligned hybrid organic/metal oxide complementary logic with megahertz operation. Org Electron.

[14] Myny K., Lai Y.-C., Papadopoulos N., Roose F.D., Ameys M., Willegems M., Smout S., Steudel S., Dehaene W., Genoe J. (2017). 15.2 A Flexible ISO14443-A Compliant 7.5mW 128b Metal-Oxide NFC Barcode Tag with Direct Clock Division Circuit from 13.56MHz Carrier. Dig Tech Pap IEEE Int. Solid State Circuits Conf..

[15] Pecunia V., Fattori M., Abdinia S., Sirringhaus H., Cantatore E. (2018). Organic and Amorphous-Metal-Oxide Flexible Analogue Electronics.

[16] Liu C.C., Wu M.L., Liu K.C., Hsiao S.-H., Chen Y.S., Lin G.-R., Huang J. (2009). Transparent ZnO Thin-Film Transistors on Glass and Plastic Substrates Using Post-Sputtering Oxygen Passivation. J. Disp. Technol..

[17] Ito M., Kon M., Miyazaki C., Ikeda N., Ishizaki M., Matsubara R., Ugajin Y., Sekine N. (2008). Amorphous oxide TFT and their applications in electrophoretic displays. Phys. Stat. Sol..

[18] Ok K.-C., Oh S., Jeong H.-J., Bae J.U., Park J.-S. (2015). Effect of Alumina Buffers on the Stability of Top-Gate Amorphous InGaZnO Thin-Film Transistors on Flexible Substrates. IEEE Electron Device Lett..

[19] Li W.-H., Shi W., Shi L.-Q., Lv X.-W., Zhang H.-J., Su C.-Y., Tseng C.-Y., Li X.-J., Liu C.-C., Wu T.-P. (2015). A 31-inch 4K2K WRGB AMOLED TV with High-Stability IGZO Back Plane. J. Soc. Inf. Disp..

[20] Ahn B.D., Jeon H.-J., Sheng J., Park J., Park J.-S. (2015). A review on the recent developments of solution processes for oxide thin film transistors. Semicond. Sci. Technol..

[21] Banger K.K., Yamashita Y., Mori K., Peterson R.L., Leedham T., Rickard J., Sirringhaus H. (2011). Low-temperature, high-performance solution-processed metal oxide thin-film transistors formed by a ‘sol–gel on chip’ process. Nat. Mater..

[22] Kim M.G., Kanatzidis M.G., Facchetti A., Marks T.J. (2011). Low-temperature fabrication of high-performance metal oxide thin-film electronics via combustion processing. Nat. Mater..

[23] Kim Y.-H., Heo J.-S., Kim T.-H., Park S., Yoon M.-H., Kim J., Oh M.S., Yi G.-R., Noh Y.-Y., Park S.K. (2012). Flexible metal-oxide devices made by room-temperature photochemical activation of sol–gel films. Nature.

[24] Yoo T.-H., Kwon S.-J., Kim H.-S., Hong J.-M., Lim J.A., Song Y.-W. (2014). Sub-second photo-annealing of solution-processed metal oxide thin-film transistors via irradiation of intensely pulsed white light. RSC Adv..

[25] Lee W.H., Lee S.J., Lim J.A., Cho J.H. (2015). Printed In-Ga-Zn-O drop-based thin-film transistors sintered using intensely pulsed white light. RSC Adv..

[26] Eom T.-Y., Ahn C.-H., Kang J.-G., Salman M.S., Lee S.-Y., Kim Y.-H., Lee H.-J., Kang C.-M., Kang C. (2018). Investigation of the evolution of nitrogen defects in flash-lamp-annealed InGaZnO films and their effects on transistor characteristics. Appl. Phys. Express..

[27] Lim H.S., Kim S.J., Jang H.W., Lim J.A. (2017). Intense pulsed light for split-second structural development of nanomaterials. J. Mater. Chem. C..

[28] Moon C.-J., Kim H.-S. (2019). Intense Pulsed Light Annealing Process of Indium-Gallium-Zinc-Oxide Semiconductors via Flash White Light Combined with Deep-UV and Near-Infrared Drying for High-Performance Thin-Film Transistors. ACS Appl. Mater. Interfaces.

[29] Kang C.-M., Kim H., Oh Y.-W., Back K.-H., Do L.-M. (2016). High-Performance, Solution-Processed Indium-Oxide TFTs Using Rapid Flash Lamp Annealing. IEEE Electron Device Lett..

[30] Jeong H.-J., Lee H.-M., Ryu C.-H., Park E.-J., Han K.-L., Hwang H.-J., Ok K.-C., Kim H.-S., Park J.-S. (2019). Ultra-High-Speed Intense Pulsed-Light Irradiation Technique for High-Performance Zinc Oxynitride Thin-Film Transistors. ACS Appl. Mater. Interfaces..

[31] Benwadih M., Coppard R., Bonrad K., Klyszcz A., Vuillaume D. (2016). High Mobility Flexible Amorphous IGZO Thin-Film Transistors with a Low Thermal Budget Ultra-Violet Pulsed Light Process. ACS Appl. Mater. Interfaces.

[32] Oh S.J., Han C.J., Kim J.W., Kim Y.-H., Park S.K., Han J.-I., Kang J.W., Oh M.S. (2011). Improving the Electrical Properties of Zinc Tin Oxide Thin Film Transistors Using Atmospheric Plasma Treatment. Electrochem. Solid-State Lett..

[33] Kwon J.Y., Jeong J.K. (2015). Recent progress in high performance and reliable n-type transition metal oxide-based thin film transistors. Semicond. Sci. Technol..

[34] Kang D., Lim H., Kim C., Song I., Park J., Pare Y. (2007). Amorphous gallium indium zinc oxide thin film transistors: Sensitive to oxygen molecules. Appl. Phys. Lett..

[35] Park J.-S., Jeong J.K., Chung H.-J., Mo Y.-G., Kim H.D. (2008). Electronic transport properties of amorphous indium-gallium-zinc oxide semiconductor upon exposure to water. Appl. Phys. Lett..

[36] Cho D.-H., Yang S., Byun C., Shin J., Ryu M.K., Park S.-H.K., Hwang C.-S., Chung S.M., Cheong W.-S., Yoon S.M. (2008). Transparent Al–Zn–Sn–O thin film transistors prepared at low temperature. Appl. Phys. Lett..

